# Drp1 overexpression induces desmin disassembling and drives kinesin-1 activation promoting mitochondrial trafficking in skeletal muscle

**DOI:** 10.1038/s41418-020-0510-7

**Published:** 2020-02-10

**Authors:** Matteo Giovarelli, Silvia Zecchini, Emanuele Martini, Massimiliano Garrè, Sara Barozzi, Michela Ripolone, Laura Napoli, Marco Coazzoli, Chiara Vantaggiato, Paulina Roux-Biejat, Davide Cervia, Claudia Moscheni, Cristiana Perrotta, Dario Parazzoli, Emilio Clementi, Clara De Palma

**Affiliations:** 1 Department of Biomedical and Clinical Sciences “Luigi Sacco”, Università degli Studi di Milano, Milano, Italy; 2 IFOM, The FIRC Institute of Molecular Oncology, Milan, Italy; 3 Neuromuscular and Rare Diseases Unit, Department of Neuroscience, Fondazione IRCCS Ca’ Granda, Ospedale Maggiore Policlinico, Milan, Italy; 4 Scientific Institute, IRCCS Eugenio Medea, Laboratory of Molecular Biology, Bosisio Parini, Italy; 5 Department for Innovation in Biological, Agro-food and Forest systems, Università degli Studi della Tuscia, Viterbo, Italy; 6 Unit of Clinical Pharmacology, ASST Fatebenefratelli Sacco and Department of Medical Biotechnology and Translational Medicine, Università degli Studi di Milano, Milano, Italy

**Keywords:** Biochemistry, Cell biology

## Abstract

Mitochondria change distribution across cells following a variety of pathophysiological stimuli. The mechanisms presiding over this redistribution are yet undefined. In a murine model overexpressing Drp1 specifically in skeletal muscle, we find marked mitochondria repositioning in muscle fibres and we demonstrate that Drp1 is involved in this process. Drp1 binds KLC1 and enhances microtubule-dependent transport of mitochondria. Drp1-KLC1 coupling triggers the displacement of KIF5B from kinesin-1 complex increasing its binding to microtubule tracks and mitochondrial transport. High levels of Drp1 exacerbate this mechanism leading to the repositioning of mitochondria closer to nuclei. The reduction of Drp1 levels decreases kinesin-1 activation and induces the partial recovery of mitochondrial distribution. Drp1 overexpression is also associated with higher cyclin-dependent kinase-1 (Cdk-1) activation that promotes the persistent phosphorylation of desmin at Ser-31 and its disassembling. Fission inhibition has a positive effect on desmin Ser-31 phosphorylation, regardless of Cdk-1 activation, suggesting that induction of both fission and Cdk-1 are required for desmin collapse. This altered desmin architecture impairs mechanotransduction and compromises mitochondrial network stability priming mitochondria transport through microtubule-dependent trafficking with a mechanism that involves the Drp1-dependent regulation of kinesin-1 complex.

## Introduction

Mitochondria play a key role in cellular homoeostasis and the proper regulation of mitochondrial transport ensures their position at sites where energy demands or oxygen supplies are the greatest [1]. Consequently, mitochondria can redistribute in response to shifts in local energy demands [2–4]. Long-range mitochondrial transport primarily involves microtubules (MT) and their specific motors (kinesins and dynein), whereas the actin cytoskeleton and its associated myosin motors support the short-range mitochondrial movement and the actin-dependent mitochondrial anchoring [5, 6].

In muscle cells, mitochondria use MT and intermediate filaments (IF) for their movement and proper localisation [7] and KIF5B mostly aids the mitochondrial transport along MT in myoblasts [8]. Conversely, IF do not provide directional transport [9] but arrest and anchor mitochondria in the specific cellular locations where they are required [10, 11]. Consistently, lack of desmin results in perturbations of mitochondrial positioning with subsarcolemmal aggregation of mitochondria [12]. The same occurs in the absence of IF–microfilament cross-linkers [11, 13, 14] supporting the evidence that IF alterations can change mitochondrial morphology and localisation.

In a mouse model overexpressing dynamin-related protein 1 (Drp1) specifically in skeletal muscle (Drp/MyoD^iCre^ (Drp/MC) mice), we found a reduction of muscle mass mainly caused by significant decrease in the weight of glycolytic muscles and a significant perturbation of mitochondrial distribution with the postnatal mitochondrial network remodelling not occurring properly and mitochondrial distribution being drastically redistributed towards the myonuclei leading to fibres with cytoplasmic region completely devoided of mitochondria [15]. Drp1 mediates mitochondrial fission [16] and its regulation is critical for myogenesis to occur [17]. A role for Drp1 in mitochondrial trafficking has never been described.

Here, we demonstrate that high Drp1 levels promote mitochondrial repositioning by activating the kinesin-1 complex. Kinesin-1 is a tetramer consisting of a homodimer of one kinesin heavy chain (KIF5A, KIF5B or KIF5C) and two kinesin light chains (KLC1 and KLC2). KIF5A and KIF5C are neuron-specific, whereas KIF5B is ubiquitously expressed [18].

We find that Drp1 binds KLC1 releasing KIF5B from kinesin-1 complex allowing its association with MT and leading to transport of mitochondria. We also demonstrate that Drp1 and fission are responsible for desmin collapse that in turn triggers the subsarcolemmal mitochondria clumping. Based on our results we propose a model whereby high Drp1 levels are responsible for desmin disruption and KIF5B-mediated transport of mitochondria involving both Cdk-1 activation and Drp1-KLC1 coupling. These mechanisms could be relevant in some human conditions, such as central core disease and myofibrillar myopathies where Drp1 is upregulated, the relevance of which also in therapeutic perspectives deserves being studied.

## Materials and methods

### Animals

All procedures involving mice were performed in accordance with the Italian law on animal care (D.L. 26/2014), as well as European Directive (2010/63/UE) and animal experimentation was approved by the Ministero della Salute (approval no. 1124/2015-PR). We used the minimal number of mice sufficient for determining significant differences in each cohort.

Mice (FBV/N C57BL/6 background) were generated as described in the previous paper [15] and were used between 7–100 days after birth compared with age-matched littermates. Mice were chosen randomly from a pool of genotyped mice at appropriate ages. Within each cohort, animals were chosen to balance the number of male and female mice in the groups and could derive from different parents. Transgenic mice were identified by PCR analysis of tail DNA using the following primers:

TgDrp1 Fw: 5′ GCA TTA CAA GGA GCC AGT CAA 3′; Rev: 5′ CAC CCT CAA AGG CAT CAC C 3′. MyoDCre Fw1: 5′ GGC TCT CTC TGC TCC TTT GA 3′; Fw2: 5′ GCG GAT CCG AAT TCG AAG TTC C 3′; Rev: 5′ TGG GTC TCC AAA GCG ACT CC 3′. Pham WT Fw 5′ CCA AAG TCG CTC TGA GTT GTT ATC 3′; Rev: 5′ GAG CGG GAG AAA TGG ATA TG 3′. Pham Mutant Fw 5′ CCC CAA CGA ATG GAT CTT G 3′; Rev: 5′ TTC GAG GGA CCT AAT AAC TTC G 3′.

Animals were maintained under controlled conditions (temperature 20 ± 2 °C; 12-hour light-dark cycle) with food and water provided *ad libitum*.

Mdivi-1 (Sigma-Aldrich, St. Louis, MO, USA) 12.5 mg/kg [19] or vehicle was intraperitoneally injected daily for 8 weeks in P25 mice. Mdivi-1 was dissolved in a 5:5:90 DMSO/cremophor/PBS vehicle and no significant differences in food and water intake were observed among the experimental groups.

For exercise experiments, mice were subjected to treadmill running at 15 m/min for 45 min [20]. Three hours after exercise, skeletal muscles (gastrocnemius) were isolated and analysed by real-time PCR.

### Human muscle samples

Central core and myofibrillar myopathy muscle bioptic tissues were provided by the Bank of muscle tissue, peripheral nerve, DNA and cell culture at Fondazione IRCCS Ca’ Granda, Ospedale Maggiore Policlinico (Milano, Italy), which is part of the Telethon Network of Genetic Biobank.

### Myotubes generation

Myotubes were obtained after satellite cells differentiation. Satellite cells (SC) were prepared from glycolytic muscles following a standardised, automated tissue dissociation protocol with a gentleMACS^™^ Octo Dissociator with Heaters (Miltenyi Biotec, Bergisch Gladbach, Germany) and magnetic depletion as previously reported [21].

To generate myotubes, SC were cultured on Matrigel-coated (BD Biosciences, San Jose, CA, USA) coverslips in DMEM (EuroClone, Pero, Milan) supplemented with 20% foetal bovine serum (EuroClone), 3% chick embryo extract (custom made), 10 ng/ml basic fibroblast growth factor (PeproTech, London, UK) and 1% penicillin-streptomycin (EuroClone) at 37 °C with 5% CO_2_ for two days to reach the sufficient cells density to promote myogenic differentiation. Myogenic differentiation was induced in DMEM supplemented with 2% horse serum (EuroClone) and 1% penicillin-streptomycin (EuroClone) and after 48 h myotubes were analysed.

For some experiments, myotubes after 24 h in differentiation medium were supplemented with Mdivi-1 1 µM [22] in DMSO and analysed after 24 h.

### Myofibre isolation and culture

Single myofibres were isolated from TA muscles of P7,P25 and P100 mice as previously described [23]. Briefly, TA muscles were dissected and incubated in DMEM (EuroClone) containing 0.2% collagenase I (Sigma-Aldrich) for 1 h. Myofibres were obtained using gentle flushing with a glass pipette and washed several times with DMEM, then they were fixed with 4% paraformaldehyde (PFA) for 10 min immediately after isolation.

### Proximity ligation assays (PLA)

Single myofibres or muscle sections were fixed (4% paraformaldehyde in PBS pH 7.5) and permeabilised (0.1% Triton X-100, 0.1M Glycine, PBS) for 10 min. Permeabilised samples were blocked with the Duolink Blocking Solution (Sigma-Aldrich) for 3 h. Primary antibodies prepared in Duolink Blocking Solution (Sigma-Aldrich) were incubated overnight at 4 °C, then PLA reactions were run using the Duolink PLA probes for mouse and rabbit and Duolink In Situ Detection Reagents Red (Sigma-Aldrich), following the manufacturer’s protocol. After some wash steps, myofibres were mounted on glass slides with Vectashield Antifade Mounting Medium with DAPI (Vector Laboratories, Burlingame, CA, USA).

Quantification of PLA was performed by counting PLA puncta per fibres and whole muscle sections. PLA experiments were performed in TA myofibres isolated from 3 different mice after which a minimum of 15 myofibres or 15 sections were analysed.

### Quantitative real-time PCR analysis

RNA was isolated from muscles using TRIzol (ThermoFisher Scientific, Waltham, MA, USA) according to already published protocols [24]. Briefly, total RNA (1 µg) was retro-transcribed using the Script Reverse Transcription Supermix (Bio-Rad, Hercules, CA, USA). RT-qPCR was performed using the g SsoAdvanced Universal SYBR Green Supermix (Bio-Rad) and the CFX96 Touch Real-Time PCR Detection System (Bio-Rad). All reactions were run as triplicates and the fold changes determined relative to the 36B4 housekeeping transcripts using the 2^−ΔΔCT^ formula [25].

For mtDNA analysis, total genomic DNA was isolated by using the QIAamp DNA Micro kit (Qiagen, Hilden, Germany). The amount of mtDNA per nuclear genome was measured by qPCR using specific primers for cytochrome b and RNase P. mtDNA quantification of the relative copy number per nuDNA was analysed using the 2^−ΔΔCT^ formula.

Below the list of primers:

Cyt b Fw: 5′ ACG CCA TTC TAC GCT CTA TC 3′; Rev: 5′ GCT TCG TTG CTT TGA GGT GT 3′. RNase P Fw: 5′ GAA GGC TCT GCG CGG ACT CG 3′; Rev: 5′ CGA GAG ACC GGA ATG GGG CCT 3′. 36B4 Fw: 5′ AGG ATA TGG GAT TCG GTC TCT TC 3′; Rev: 5′ TCA TCC TGC TTA AGT GAA CAA ACT 3′. PGC-1α Fw: 5′ TCT GAG TCT GTA TGG AGT GAC AT 3′; Rev: 5′ CCA AGT CGT TCA CAT CTA GTT CA 3′. Slc3a2 Fw: 5′ GGG GAG CGT ACT GAA TCC CT 3′; Rev: 5′ CTG AAG GCC AAG CTC ATC CC 3′. Slc6a9 Fw: 5′ GTT GGC GCT TTG TTT CTC CG 3′; Rev: 5′ TCT GCT TGG CTT TGT GGC AT 3′. Mthfd2 Fw: 5′ CAT GGG GCG TGT GGG AGA TAA T 3′; Rev: 5′ CCG GGC CGT TCG TGA GC 3′. Asns Fw: 5′ ATT ACG ACA GTT CGG GCA TC 3′; Rev: 5′ TCT CAG TTC GAG ACC GTG TG 3′. Gadd45a Fw: 5′ CGG TGA TGG CAT CCG AAT GGA AAT 3′; Rev: 5′ TCT GCA AAG TCA TCT CTG AGC CCT 3′. P21 Fw: 5′ CAA AGT GTG CCG TTG TCT CT 3′; Rev: 5′ GTC AAA GTT CCA CCG TTC TC 3′. KLC1 Fw: 5′ AGC GGG AGT TTG GAT CTG TG 3′; Rev: 5′ AGC CAC TCT CTG CTT ACG TGA 3′. KIF5B Fw: 5′ GCG GAG TGC AAC ATC AAA GTG 3′; Rev: 5′ CAT AAG GCT TGG ACG CGA TCA 3′.

### Immunoblotting

Proteins were extracted from muscles and myotubes. Muscles were homogenised in a lysis buffer containing 20 mM Tris-HCl (pH 7.4), 10 mM EGTA, 150 mM NaCl, 1% Triton X-100 (Sigma-Aldrich, X100), 10% glycerol, 1% SDS (Sigma-Aldrich, L3771) supplemented with a cocktail of protease and phosphatase inhibitors (cOmplete and PhosSTOP; Sigma-Aldrich) with an Ultra-Turrax (Ika Werke GmbH & Co). Myotubes lysis was performed in the RIPA buffer (50 mM Tris-HCl, pH 7.4, 150 mM NaCl, 0.1% SDS (Sigma-Aldrich), 1% NP-40 (Sigma-Aldrich), 0.25% sodium deoxycholate (Sigma-Aldrich), 1 mM EDTA) supplemented with protease and phosphatase inhibitors. After centrifugation at 10,000 × *g* for 10 min, proteins were quantified with the Bio-Rad protein assay after which 30–50 µg were loaded on 4–20% polyacrylamide precast gels (Criterion TGX Stain-free precast gels; Bio-Rad). Proteins were transferred onto a nitrocellulose membrane using a Trans-Blot Turbo SystemTM (Bio-Rad) and Transfer packTM (Bio-Rad). The revelation was carried out by specific horseradish peroxidase-labelled secondary antibodies (Bio-Rad), followed by chemiluminescent detection (Bio-Rad) with the ChemiDoc MP imaging system (Bio-Rad). The blots were routinely treated with glycine (0.2 M pH 2.5) stripping buffer and reprobed.

For the preparation of extracts to detect desmin and desmin tetramers, fresh TA muscles were dissected and homogenised with an Ultra-Turrax in ice-cold extraction buffer containing 50 mM Tris–HCl pH 8, 50 mM NaCl, 5 mM EDTA, 5 mM EGTA, 0.5% NP‐40, 0.05% SDS. After 30 min of incubation on ice, the extracts were centrifuged for 30 min at 30,000 rpm at 4 °C. After centrifugation, pellets were freeze-thawed twice, homogenised in a small volume and centrifuged for 30 min at 30,000 rpm at 4 °C. Finally, the supernatants were pooled and quantified for immunoblotting.

### Immunoprecipitation

For immunoprecipitation experiments, muscles were homogenised with an Ultra-Turrax (Ika Werke GmbH & Co, Staufen, Germany) in a lysis buffer containing 20 mM Tris–HCl pH 8, 137 mM NaCl, 10% Glycerol, 1% NP‐40, 2 mM EDTA, protease and phosphatase inhibitor cocktail (cOmplete and PhosSTOP; Sigma-Aldrich). Lysates were cleared by centrifugation and the same amount of proteins were incubated overnight with either the specific primary antibodies and the appropriate protein A or protein G Sepharose (GE Healthcare, Chicago, IL, USA) beads. Beads were washed three times in lysis buffer and eluted in Laemmli buffer. The whole‐lysate (input) and the immunoprecipitated (IP) samples were separated by SDS–PAGE, transferred to nitrocellulose membranes, incubated with the proper antibodies, and developed according to standard procedures described above.

### Immunofluorescence

Muscles were dissected and immediately frozen in liquid N_2_-cooled isopentane (Sigma-Aldrich) to allow the preparation of 5 μm thick sections, while single fibres were prepared as described above.

For immunofluorescence, we followed a standard protocol [26] for both muscle sections and single fibres. In brief, samples were fixed with PFA (Sigma-Aldrich) 4% for 10 min and permeabilised with 0.1% TritonX-100 (Sigma-Aldrich) in PBS (EuroClone). Sections were then blocked for 30 min in blocking buffer containing 2% normal goat serum (NGS; Vector Laboratories), 0.5 % bovine serum albumin (BSA) and PBS (EuroClone). All primary antibodies were diluted in blocking buffer and incubated at room temperature for 2 h. Samples were subsequently washed three times with PBS and incubated with fluorophore-conjugate (Alexa Fluor conjugates, ThermoFisher) secondary antibodies for 1 h at room temperature after which nuclei were counterstained with DAPI (ThermoFisher Scientific). Slides were finally mounted with the ProLong Gold antifade reagent (ThermoFisher Scientific).

### Antibodies and reagents

The following antibodies were used: antibodies against Drp1 (D6C7) (#8570S; WB 1:1000, IF 1:100, IP/PLA 1:100), P-Drp1(S616) (#3455S; WB 1:1000), P-Drp1 (S637) (#4867S; WB 1:1000), Desmin (D93F5) (5332S; WB 1:1000, IF 1:100), eIF2alpha (#9722S; WB 1:1000) and P-eIF2alpha (S51) (#9721; WB 1:1000), Cdc2 (#28439; WB 1:1000) and P-Cdc2 (Thr161) (#9114; WB 1:1000) which were purchased from Cell Signaling Technology (Beverly, MA, USA); antibodies against KLC1 (L2) (sc-58776; WB 1:500 IF 1:100) and GAPDH (FL-335) (sc-25778; WB 1:3000) which were purchased from Santa Cruz Biotechnology (Dallas, TX, USA); antibodies against alpha-Tubulin (ab11304-100; IF/PLA 1:100), KIF5B (ab167429; WB 1:1000, IF/PLA 1:200, IP 1:100), and KLC1 (ab174273; IF/PLA 1:100) which were purchased from Abcam (Cambridge, UK); an antibody against DLP1 (i.e. Drp1) (611113: WB 1:800) which was purchased from BD Biosciences; antibodies against Desmin (D1033; WB 1:500) and Clpp (WH0008192M1; WB: 1:1000) which were purchased from Sigma-Aldrich; an antibody against OxPhos Complex IV subunit IV (i.e. COX IV) (20E8C12; WB 1:2000, IF 1:50) which was purchased from Invitrogen (Carlsbad, CA); the antibody against p-desmin Ser-31 (#D375-3; WB 1:1000, IF 1:500) was purchased from MBL Life Science (Woburn, MA, USA).

### Microtubules spin-down assay

MT binding assay was performed using the MT-binding protein spin-down assay kit (Cytoskeleton, Denver, CO, USA) following the manufacturer’s instructions. For protein extracts, fresh gastrocnemius muscles were homogenised using an Ultra-Turrax in a PIPES-based buffer (80 mM PIPES, 1 mM EGTA, 1 mM MgCl_2_, 0.3% Triton X-100, 10% glycerol, supplemented with a cocktail of protease and phosphatase inhibitors). For the spin-down procedure, in vitro polymerised MT were incubated for 30 min at room temperature with 8 μg of purified MAP2, 3 μg of BSA or 100 μg of the muscle protein extracts described above. As a control, the proteins were incubated in the absence of MT. MT (pellet fraction: P) and soluble proteins (supernatant fraction: S) were then separated by ultra-centrifugation at 100,000 × *g* at room temperature for 40 min using a cushion buffer. To remove the high amount of tubulin, pelleted fractions were incubated with the Salt Extraction Buffer provided by the manufacturer following ultra-centrifugation at 100,000 × *g* at room temperature for 40 min for the salting-off procedure; this step ensures the dissociation between MT and MT-binding proteins. Subsequently, the proteins were precipitated via a trichloroacetic acid (TCA) extraction: the TCA solution (1:10) was added to the samples and incubated 15 min on ice. Following centrifugation at 14,000 rpm for 10 min at 4 °C the pellet was resuspended in 1 M Tris-base, pH 10.4. S and purified P fractions were loaded onto 4–20% polyacrylamide precast gels and analysed for the presence of KIF5B, Drp1 and KLC1 by immunoblotting.

### Image acquisition and analysis

#### Confocal imaging

Confocal images were acquired on a Leica TCS SP8 System equipped with a Leica DMi8 inverted microscope, using a Leica HC PL APO CS2 40X/1.30 oil immersion objective. Samples were illuminated with 405 nm laser line for the detection of DAPI signal and 561 nm tuned with light laser for the detection of both Cy3 signal and DIC image. The acquisition software used was the Leica Application Suite X, ver. 3.5.2.18963 (Leica, Wetzlar, Germany). An image format of 1024 × 1024 pixel was used and both optical zoom ×1 and ×2 were used for the samples acquisition.

For three-dimensional reconstruction of mitochondria, 0.5 μm thick Z-stacks were acquired using a Leica TCS SP8 System equipped with a Leica DMi8 inverted microscope; three-dimensional projections were generated using the Leica LAS X software.

#### Time-lapse imaging

Time-lapse imaging was performed on myotubes and muscle fibres.

For myotubes, time-lapse movies were recorded using a Confocal Spinning Disk microscope (Olympus, Shinjuku, Tokyo, Japan) equipped with an IX83 inverted microscope provided with an IXON 897 Ultra camera (Andor, Belfast, UK), using a 60× UPlanSApo 1.35NA objective. The system is driven by the Olympus CellSens Dimension 1.18 software (Build 16686). Temperature, CO_2_ concentration and humidity were controlled using an OKOlab incubator. FITC signals were excited with a 488 nm 50 mW diode lasers. Eight or 10 min time-lapse movies were recorded with a 10 s time-frame setting.

Time-lapse movies on muscles were recorded using an UltraVIEW VoX spinning-disk confocal system (PerkinElmer, Waltham, MA, USA) equipped with an EclipseTi inverted microscope (Nikon, Minato, Tokyo, Japan) provided with a Yokogawa CSU-X1 confocal scanner unit, an integrated FRAP PhotoKinesis module (PerkinElmer), a Hamamatsu CCD camera (C9100-50) and driven by the Volocity software ver. 6.3.1 (Improvision; Perkin Elmer). All images were acquired through a 60× oil-immersion objective (Nikon Plan Apo VC, NA 1.4). Temperature, CO_2_ concentration and humidity were controlled using an OKOlab incubator. Ten minutes time-lapse movies were recorded with a 2 s time-frame setting.

The photoconversion experiments on muscle were performed using the 405 nm laser. GFP and mCherry signals were excited respectively with a 488 nm and 561 nm 50 mW diode lasers.

#### Mitochondria morphometric analysis

An Image J macro was used to quantify mitochondria morphology [27, 28]. The green channel of PhAM myotubes was extracted to grayscale, thresholded to resolve optimally individual mitochondria and converted to a binary image. For the analysis of mitochondrial branching, the binary image was converted to a skeleton by using “skeletonise”. Finally, the length of each branch and the number of branches were determined by using the “analyse skeleton” plugin.

#### Analysis of mitochondrial distance travelled

Time-lapse sequences were imported into ImageJ after which individual mitochondria were manually tracked using the Manual Tracking plugin. Mitochondria were tracked along the stacks and the distance between frames was used to calculate the cumulative distance travelled by each individual mitochondria. At least ten mitochondria for myotubes were tracked and the experiment was repeated three times.

#### Mitochondrial displacement index

Mitochondrial displacement was quantified by calculating the ratio between the area of the maximum projection of the fluorescence intensity and the area of the fluorescence intensity of the mitochondria network on the first frame.

The obtained ratio represents the fraction of mitochondrial mass that changed position over time. We developed a custom Fiji Plugin [29] to obtain the two areas and their relative ratios. Data were obtained from three independent experiments.

#### MyToe analysis

Maps of the instantaneous mitochondrial velocities were obtained by analysing time-lapse movies (on the FITC channel) and performing an Optical Flow analysis using a Mytoe Software on pre-selected cell regions [30].

The instantaneous rootmeans square velocity *V*_r.m.s_(*t*) of a single region for each time point *t*, was calculated as

$$V_{{\mathrm{r}}.{\mathrm{m}}.{\mathrm{s}}.}\left( t \right) \,=\, \frac{1}{{N_{\mathrm{valid}}}}\sqrt {\mathop {\sum }\limits_{n \,=\, 1}^{N_{\mathrm{valid}}} |{\boldsymbol{v}}\left( {{\boldsymbol{x}}_n,t} \right)|^2},$$

where *N*_valid_ is the number of grid points in the mitochondrial region (obtained automatically from the software) and ${\boldsymbol{v}}\left( {{\boldsymbol{x}}_n,t} \right)$ is the instantaneous velocity at the *n*th grid point ***x***_*n*_ with $|{\boldsymbol{v}}\left( {{\boldsymbol{x}}_n,t} \right)|^2$ > 7 nm/s.

The analysis was performed on three independent experiments per condition.

### Statistics

We have not used a statistical method to predetermine samples size. Sample size was chosen according to our experience with biochemical and in vivo experiments and no samples were excluded from the analysis. The experiments were not carried out in blinding conditions and the results were shown as mean ± SEM if not otherwise indicated. A GraphPad Prism (GraphPad Software, Inc) was used to analyse the data. The D’Agostino and Pearson omnibus test was applied to assess normal distribution of data. Comparison of two groups normally distributed was performed using an unpaired two-tailed *t*-test while non-normally distributed data were analysed with the Mann–Whitney test. Comparison of multiple groups was performed by one-way ANOVA followed by post hoc Tukey’s test. For grouped analyses, two-way ANOVA with correction for multiple comparisons using the Holm–Sidak method was used. A *P* value < 0.05 was considered significant.

## Results

### Drp1 is more active in Drp/MC mice leading to progressive alterations of mitochondrial network

Overexpression of Drp1 in Drp/MC mice affects the normal remodelling of the mitochondrial network by redistributing mitochondria closer to the sarcolemma and/or myonuclei [15]. A mild defect is present already in growing muscle starting at P7 (P, postnatal day) to become more evident in the adult muscle at P100.

**Fig. 1:**
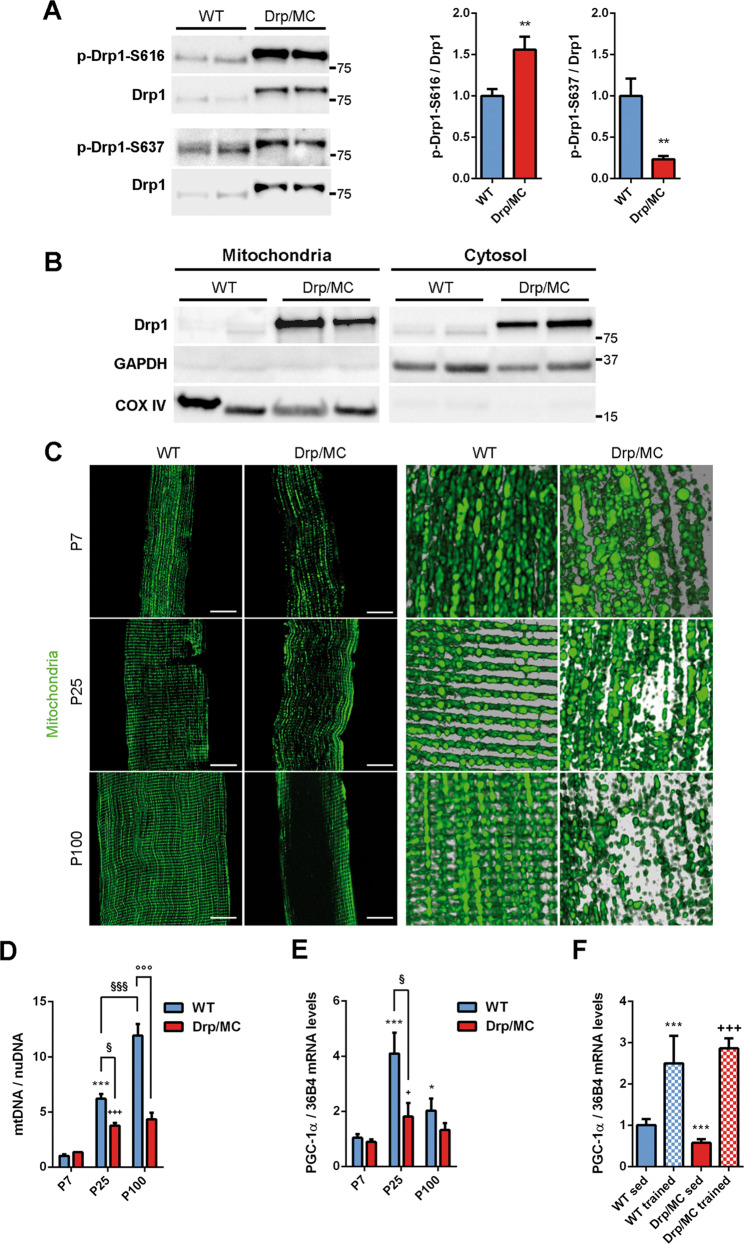
Drp1 overexpression impacts on mitochondrial content, positioning and remodelling. **a** Representative immunoblotting of phospho-Drp1 on serine 616 (p-Drp1-S616) and serine 637 (p-Drp1-S637) and total Drp1 in Tibialis Anterior (TA) lysates from WT and Drp/MC mice at P25. Phosphorylated Drp1 levels normalised on total Drp1 were quantified and shown as fold change of WT (*n* ≥ 7 mice per genotype). * vs WT (**P < 0.01). **b** Representative immunoblotting of Drp1 in mitochondrial and cytosolic fractions of TA from WT and Drp/MC mice at P25. COX IV and GAPDH have been used as mitochondrial and cytosolic markers respectively. **c** Confocal images (left) and magnified 3D reconstructions (right) of TA muscle fibres from WT/PhAM and Drp/MC/PhAM at P7, P25 and P100 showing mitochondrial network (green) (scale bar = 10 µm). **d** Quantitative PCR (qPCR) analysis of mtDNA content in TA from WT and Drp/MC mice at P7, P25 and P100. DNA levels were quantified and shown as fold change of P7 WT (*n* ≥ 3 mice per genotype). * vs WT at P7 (***P < 0.001); + vs Drp/MC at P7 (^+++^*P* < 0.001); § vs WT at P25 (^§^*P* < 0.05, ^§§§^*P* < 0.001); ° vs WT at P100 (^°°°^*P* < 0.001). **e** Quantitative Reverse Transcription PCR (RT-qPCR) analysis of PGC-1α in gastrocnemius from WT and Drp/MC mice at P7, P25 and P100. Data are shown as fold change of P7 WT (*n* ≥ 4 mice per genotype). * vs WT at P7 (*P < 0.05, ***P < 0.001); +vs Drp/MC at P7 (+*P* < 0.05); ^§^ vs WT at P25 (^§^*P* < 0.05). **f** RT-qPCR analysis of PGC-1α in gastrocnemius from WT and Drp/MC mice at P100 either sedentary or trained. Data are shown as fold change of sedentary WT (*n* ≥ 3 mice per condition). * vs sedentary WT (***P < 0.001); +vs sedentary Drp/MC (^+++^*P* < 0.001). Values are expressed as mean ± SEM.

The balance between Drp1 Ser-616/Ser-637 phosphorylation reflects Drp1 activity [31, 32]. In Drp/MC transgenic mice we found a significant increase in Drp1 phosphorylation at Ser-616 combined with a decreased phosphorylation at Ser-637, indicating a hyperactivation of Drp1 (Fig. 1a). This was paralleled by an increased association of Drp1 with mitochondria suggesting a pro-fission state of mitochondria. Despite this, mitochondrial Drp1 localisation was only partial as clearly shown by its high residual levels in the cytosolic fraction (Fig. 1b).

To analyse further this aspect we took advantage of mice with photo-activatable mitochondria (PhAM mice) which were crossed with wild-type (WT) and Drp/MC mice [15] and allowed us to perform a 3D reconstruction of the mitochondrial network in single fibres. Normal fibres reported the typical striated distribution of intermyofibrillar and subsarcolemmal mitochondria (Fig. 1c, left panels), whereas Drp1-overexpressing fibres from an early stage of development (P7) showed a disorganised mitochondrial network characterised by isolated ellipsoid‐shaped mitochondria (Fig. 1c, upper panels). At P25 an initial redistribution of fissioned mitochondria towards the sarcolemma became apparent (Fig. 1c, middle panels) and at P100, regions of cytoplasm completely devoided of mitochondria were observed (Fig. 1c, lower panels).

In Drp/MC mice the postnatal mitochondrial biogenesis occurred to a lesser extent compared with WT and after P25 the mitochondrial content in Drp/MC mice did not change further (Fig. 1d). By contrast, mitochondrial DNA (mtDNA) levels increased progressively with age in WT mice (Fig. 1d). Consistently, peroxisome proliferator-activated receptor-gamma coactivator 1 α (PGC-1α), the master regulator of mitochondrial biogenesis [33], had a peak of expression at P25 in WT mice that was milder in Drp/MC mice (Fig. 1e), confirming a reduced induction of mitochondrial biogenesis during muscle postnatal growth. Conversely, after a bout of forced exercise, both WT and transgenic mice upregulated PGC-1α without differences between genotypes (Fig. 1f), indicating that the mitochondrial biogenesis machinery is not affected in Drp/MC mice, rather, it is defectively stimulated by an impaired postnatal muscle growth [15]. This finding, alongside with the mitochondrial repositioning we observed, is not surprising as mitochondria are transported to optimise ATP delivery [34]. We thus investigated whether Drp1 was responsible for the mitochondrial repositioning in Drp/MC mice.

### Drp1 regulates microtubule-dependent mitochondrial trafficking

Mitochondria primarily move along MT tracks, an action that requires their coupling with motor proteins [18]. As muscle cells require the kinesin-1 complex for mitochondrial movements [8] we investigated its role in Drp/MC muscle. At P1 and P7, when the mitochondrial trafficking was absent, the levels of KIF5B and KLC1 were unchanged in Drp/MC muscle (Fig. S1A). However, at P25, when the repositioning of mitochondria was at its beginning, KIF5B and KLC1 protein levels were efficiently upregulated in Drp/MC muscles (Fig. S1B). Conversely, at P100, when the repositioning of mitochondria had already occurred, only KIF5B was still higher; by contrast, the levels of KLC1 were similar to those observed in WT (Fig. S1C). Considering that KIF5B and KLC1 expressions were similar in WT and Drp/MC mice at both P25 and P100 (Fig. S1D), their increase appears consistent with translation or post-translation regulation.

**Fig. 2:**
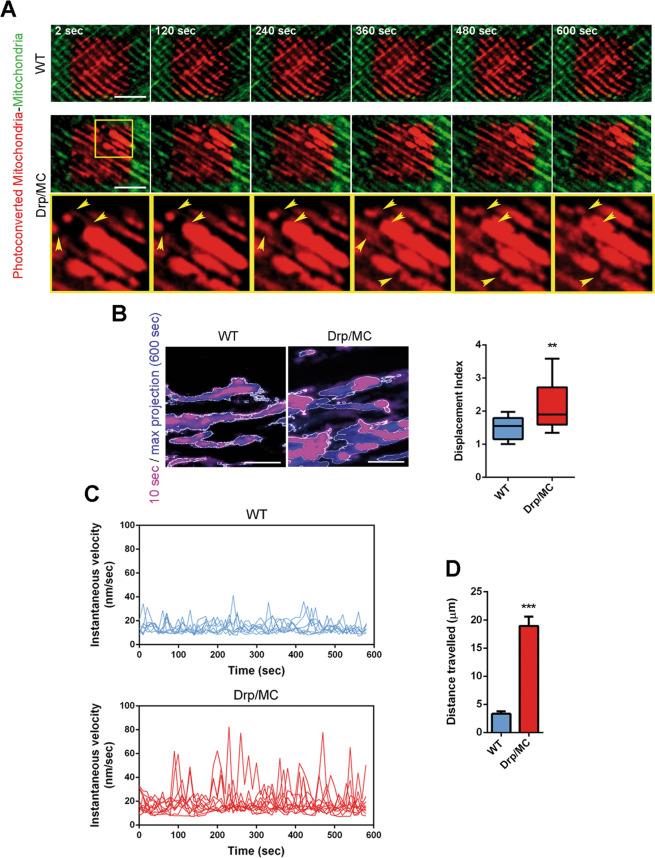
Drp1 overexpression increases mitochondrial movements. **a** Stills from the movie showing mobility of WT/PhAM and Drp/MC/PhAM mitochondria (TA). Arrowheads indicate mitochondrial movements during the time (scale bar = 5 µm). **b** Stills from movies showing the area occupied by mitochondria in WT and Drp/MC myotubes at  10 s (magenta) and after 600 s (blue) (scale bar = 5 µm). Displacement Index was calculated (*n* ≥ 3 independent experiments). * vs WT (**P < 0.01). **c** Instantaneous velocities of mitochondria in selected ROI were determined in WT and Drp/MC myotubes using Mytoe software (*n* ≥ 3 independent experiments). **d** Distance travelled by mitochondria in WT and Drp/MC myotubes calculated by ImageJ software (*n* ≥ 3 independent experiments *n* ≥ 20 mitochondria). * vs WT (***P < 0.001). Values are expressed as mean ± SEM.

In agreement with the increased amount of kinesin-1 complex, at P25 mitochondria in Drp/MC/PhAM fibres exhibited a faster speed of movements with evident changes in mitochondrial position over a 10 min period as assessed by time-lapse imaging experiments in muscle fibres after mitochondria photoconversion (Fig. 2a and Video 1).

To assess further the role of Drp1 overexpression in the mitochondrial movement we used myotubes derived from primary myoblasts isolated by sorting from WT/PhAM and Drp/MC/PhAM muscles. In agreement with the results obtained in fibres, we observed a disorganised and fragmented mitochondrial network, suggesting that myotubes could mimic in vivo condition (Fig. S2A). Drp1 overexpression significantly increased mitochondrial displacement index measured as the fraction of mitochondrial mass that changed position between frames during the time [35, 36] (Fig. 2b and Video 2). Therefore, mitochondria in Drp-overexpressed myoblasts displayed high capability of redistribution during a short period (Fig. S2B). They also exhibited high fluctuations in instantaneous velocity, measured through Mytoe software [30], reaching a maximum of 80 nm/sec, whereas the instantaneous velocities in WT myotubes were lower and relatively constant (Fig. 2c).

Mitochondrial movement is mediated by the actin cytoskeleton for short-range displacements and the MT cytoskeleton for longer range movements [35]. In muscle, MT form a non-classic grid-like network [37] that was unaffected by Drp1 overexpression (Fig. S3A, B) as well as the actin microfilaments (MF) (Fig. S3A, B), confirming the possibility for mitochondria to move along the cytoskeleton. Consistently, we detected a directional displacement of mitochondria in Drp/MC myotubes covering a longer distance (µm) over a 10 min period compared with WT myotubes (Fig. 2d, Fig. S3C and Video 3), indicating a boosted MT-mediated transport of mitochondria.

### Drp1 sequesters KLC1 allowing transport of mitochondria by KIF5B

**Fig. 3:**
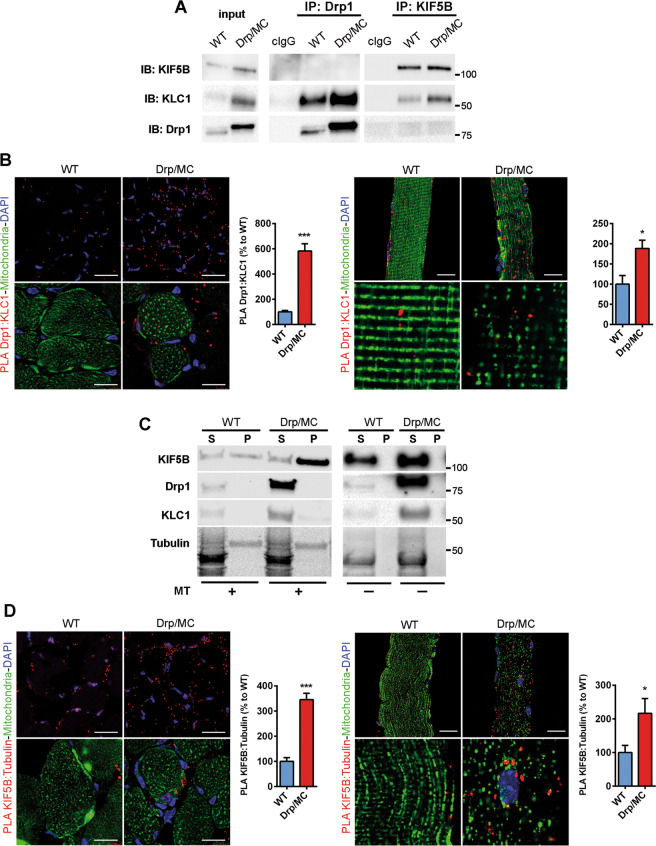
Drp1 binds KLC1 and affects kinesin-1 activation. **a** Co-immunoprecipitation of Drp1, KIF5B and KLC1 in TA lysates from WT and Drp/MC mice at P25. Control immunoglobulins (cIgG) were used as IP negative control. **b** Representative Drp1:KLC1 PLA (red puncta) on TA sections (left panel) and single fibres (right panel) from WT/PhAM and Drp/MC/PhAM (scale bar = 25 µm for both section and fibre). COX4-FITC (green) and DAPI (blue) signals (scale bar = 20 µm) and PhAM fibre magnification are provided. PLA puncta were quantified and shown as a percentage relative to WT (*n* = 3 mice per genotype). **c** Microtubules (MT) spin-down assay using TA lysates from WT and Drp/MC mice at P25 incubated with (+) or without (−) polymerised Tubulin. Pellet (P) and supernatant fractions (S) were analyzed by immunoblotting for KIF5B, Drp1 and KLC1. After salting-off procedure, detectable tubulin residues are still visible in P fractions (stain-free gel). **d** Representative KIF5B:α-Tubulin PLA (red puncta) on TA sections (left panel) and single fibres (right panel) from WT/PhAM and Drp/MC/PhAM (scale bar = 25 µm for both section and fibre). COX4-FITC (green) and DAPI (blue) signals (scale bar = 20 µm) and PhAM fibre magnification are provided. PLA puncta were quantified and shown as a percentage relative to WT (*n* = 3 mice per genotype). Values are expressed as mean ± SEM. * vs WT (*P < 0.05, ***P < 0.001).

The enhanced directional mitochondrial movements in the presence of high levels of Drp1 would suggest its involvement in the regulation of the kinesin-1 complex. Drp1 has been recognised as a novel binding partner for KLC1 [38]. In Drp/MC muscle homogenates, Drp1 clearly pulled down KLC1, but not KIF5B, indicating that KIF5B is not in the same complex with Drp1 and KLC1 (Fig. 3a). Similarly, KIF5B did not pull down Drp1 (Fig. 3a), confirming that the Drp1-KLC1 complex is formed regardless of KIF5B.

To confirm the specificity of Drp1 and KLC1 binding, we performed the Drp1:KLC1 proximity ligation assay (PLA) on muscle sections and single muscle fibres and we detected enhanced PLA signal in Drp/MC mice (Fig. 3b). Of note, PLA puncta did not colocalise with mitochondria indicating that only the cytosolic fraction of Drp1 was able to interact with KLC1 (Fig. 3b). The involvement of cytosolic Drp1 in Drp1-KLC1 complex was further supported by fractionation experiments, in which KLC1 was found only in the cytosol (Fig. S3D) while Drp1 could be detected in both the mitochondrial and cytosolic fractions (Fig. 1b).

Transport of mitochondria along the axon may not require KLC1 [39]. Whether this is the case also in muscle it is unknown. To explore this issue we assessed the amount of kinesin-1 in the active state, looking at the components of the complex associated with MT [40]. To this end, we performed the MT spin-down assay with WT and Drp/MC muscle homogenates. Only KIF5B co-sedimented with MT while Drp1 and KLC1 were enriched in the unbound fraction (Fig. 3c), confirming that the active form of kinesin-1 involves only its heavy chain KIF5B, but not the tetramer. Moreover, kinesin-1 was more active in Drp1 overexpressing muscle, where more KIF5B co-sedimented with MT.

In agreement with this evidence, the KIF5B:α-tubulin PLA signal was higher in Drp/MC sections and single fibres when compared with those of the WT (Fig. 3d); otherwise no signal was detected for KLC1:α-tubulin PLA (Fig. S3E), confirming that the active binding of KIF5B on MT does not require KLC1 and that kinesin-1 is more active in Drp1 overexpressing muscles.

**Fig. 4:**
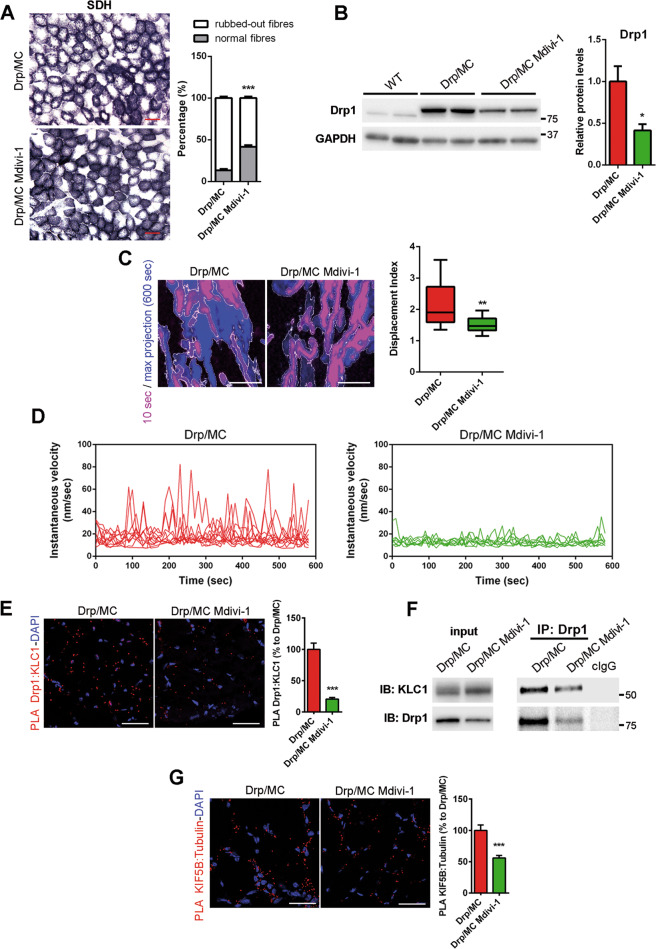
Mdivi-1 treatment reduces Drp1-KLC1 binding and mitochondrial movements. **a** Succinate dehydrogenase (SDH) staining of TA muscle sections from Drp/MC mice treated or not with Mdivi-1 (12.5 mg/kg). Percentage of rubbed-out fibres were quantified and shown as percentage of total fibres (*n* = 3 mice per condition) (scale bar = 50 µm). **b** Representative immunoblotting of Drp1 and GAPDH as loading control in TA lysates from WT and Drp/MC mice treated or not with Mdivi-1. (*n* ≥ 3 mice per condition). **c** Stills from movies showing the area occupied by mitochondria from Drp/MC myotubes treated or not with Mdivi-1 at  10 s (magenta) and after 600 s (blue) (scale bar = 5 µm). Displacement Index was calculated (*n* ≥ 3 independent experiments). * vs untreated Drp/MC myotubes (**P < 0.01). **d** Instantaneous velocities of mitochondria in selected ROI were determined in Drp/MC myotubes treated or not with Mdivi-1 using Mytoe software (*n* ≥ 3 independent experiments). **e** Representative PLA Drp1:KLC1 (red puncta) on TA muscle sections from Drp/MC mice treated or not with Mdivi-1. DAPI nuclear counterstaining (blue) is provided (scale bar = 25 µm). PLA puncta were quantified and shown as a percentage relative to untreated Drp/MC mice (*n* = 3 mice per condition). **f** Co-immunoprecipitation of Drp1 and KLC1 in gastrocnemius lysates from Drp/MC mice treated or not with Mdivi-1. Control immunoglobulins (cIgG) were used as IP negative control. **g** Representative PLA KIF5B:α-Tubulin (red puncta) on TA muscle sections from Drp/MC mice treated or not with Mdivi-1. DAPI nuclear counterstaining (blue) is provided (scale bar = 25 µm). PLA puncta were quantified and shown as a percentage relative to untreated Drp/MC mice (*n* = 3 mice per condition). Values are expressed as mean ± SEM. * vs untreated Drp/MC mice (*P < 0.05, **P < 0.01, ***P < 0.001).

To assess whether Drp1 is involved in the activation of kinesin-1 complex, we treated Drp/MC mice at P25 with a daily intraperitoneal injection of the Drp1 inhibitor Mdivi-1 for 8 weeks. In Drp/MC mice Mdivi-1 induced a 40% recovery of fibres with normal SDH staining (Fig. 4a) accompanied by a more organised mitochondrial network without region devoided of mitochondria (Fig. S4A). Moreover, Mdivi-1 increased mitochondrial branch length compared with untreated Drp1/MC mice (Fig. S4B). These changes were not coupled to mtDNA recovery (Fig. S4C) supporting a role of Drp1 only in the maintenance of normal mitochondria positioning and in the regulation of mitochondrial trafficking. Accordingly to Manczak et al. [41], we found a significant Mdivi-1-dependent drop of Drp1 levels in muscle (Fig. 4b). By contrast, Mdivi-1 did not reduce the expression of both KIF5B and KLC1 (Fig. S4D). We confirmed these findings in myotubes treated for 24 h with Mdivi-1 (1 µM) [22]. As expected, Mdivi-1 reversed the defect in the mitochondrial network in Drp1 overexpressing myotubes increasing both the mitochondrial branches and the branch length (Fig. S4E). We also found, accordingly to muscle, a significant reduction of Drp1 levels in myotubes and an unchanged expression of KIF5B and KLC1 (Fig. S4F). Consistently, Mdivi-1 reduced the motile properties of mitochondria in Drp/MC myotubes with respect to their net displacement (Fig. 4c and Video 4) and the fluctuation of their instantaneous velocity (Fig. 4d).

Comparable to the 50% decrease in Drp1 levels, we found a proportional reduction in Drp1:KLC1 PLA signal in muscle sections (Fig. 4e) and fibres (Fig. S4G) of Drp/MC mice treated with Mdivi-1, as well as a proportional reduction in the amount of KLC1 co-immunoprecipitated with Drp1 in Mdivi-1-treated muscle homogenates (Fig. 4f). Both findings indicate that KLC1 is less sequestered by Drp1 and likely more associated with KIF5B. Strikingly, the KIF5B:α-tubulin PLA interactions were reduced after Mdivi-1 exposure, confirming the presence of a less active kinesin-1 (Fig. 4g and Fig. S4H).

Taken together, these data provide compelling evidence that in Drp/MC mice cytosolic Drp1 binds KLC1, leading to dissociation of the kinesin-1 tetramer and allowing KIF5B to associate with MT to transport mitochondria.

### Drp1 can affect desmin assembling promoting mitochondrial repositioning

**Fig. 5:**
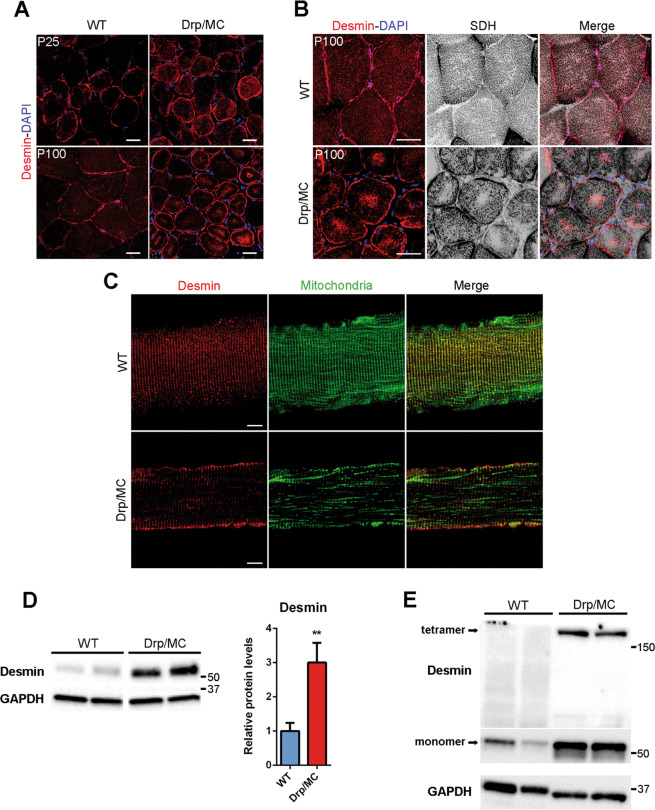
Defective desmin network in Drp/MC mice. **a** Representative desmin immunostaining (red) of TA sections from WT and Drp/MC mice at P25 and P100. DAPI is used as nuclear staining (blue) (scale bar = 25 μm). **b** Representative desmin immunostaining (red) and SDH staining (grey) of TA sections from WT and Drp/MC mice at P100. DAPI is used as nuclear staining (blue) (scale bar = 25 μm). **c** Representative desmin immunostaining (red) of TA fibres from WT/PhAM and Drp/MC/PhAM mice at P100. Mitochondrial network (green) images  are provided (scale bar = 10 μm). **d** Representative immunoblotting of desmin in TA lysates from WT and Drp/MC mice at P100. GAPDH has been used as a loading control. Desmin levels were quantified and shown as fold change of WT (*n* ≥ 6 mice per genotype). **e** Representative immunoblotting showing the 200-kDa bands detected by the desmin antibody corresponding to a detergent-resistant tetramers in TA lysates from WT and Drp/MC mice at P100. GAPDH has been used as a loading control. Values are expressed as mean ± SEM. * vs WT (**P < 0.01).

MT and MF networks were unaffected in Drp/MC mice, however, the proper localisation of mitochondria is also maintained by desmin IF; hence we explored their morphology in Drp/MC mice. Muscle transversal sections of WT mice showed that desmin IF were typically located underneath the sarcolemma and at the level of Z-discs (Fig. 5a, left panel), however, at P25 in Drp/MC mice some desmin aggregates were found in the subsarcolemmal region (Fig. 5a upper panels). This phenotype worsened with age such that, in Drp/MC mice at P100, desmin was massively accumulated in the subsarcolemmal region and formed sarcoplasmic aggregates in most fibres (Fig. 5a lower panel).

We also performed SDH staining and desmin immunofluorescence which showed that sarcoplasmic desmin aggregates were in mitochondria rubbed-out areas (Fig. 5b).

This desmin collapse was confirmed in single fibres preparation. At P100, while WT fibres did not show any overt abnormalities, substantial changes in the desmin pattern were found in Drp/MC/PhAM fibres (Fig. 5c). At P25 the Drp/MC/PhAM phenotype was milder, displaying only partial accumulation of desmin beneath the sarcolemma (Fig. S5A).

**Fig. 6:**
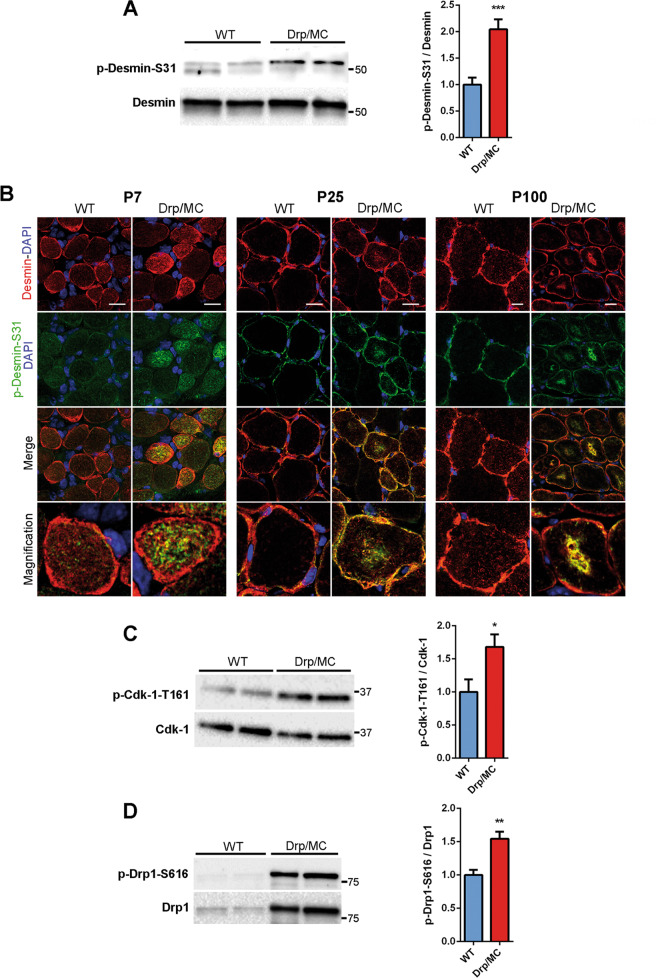
Cdk-1 activation drives desmin phosphorylation at Ser-31 resulting in its disassembling. **a** Representative immunoblotting of phospho-desmin on serine-31 (p-Desmin-S31) in gastrocnemius lysates from WT and Drp/MC mice at P25. Total desmin has been used as loading control. Phospho-desmin levels normalised on total desmin were quantified and shown as fold change of WT (*n* = 8 mice per genotype). **b** Representative p-Desmin-S31 (green) and desmin (red) immunostainings of TA sections from WT and Drp/MC mice at P7, P25 and P100. DAPI is used as nuclear staining (blue) (scale bar = 10 μm). **c** Representative immunoblotting of phospho-Cdk-1 on Threonine 161 (p-Cdk-1-T161) in gastrocnemius lysates from WT and Drp/MC mice at P7. Total Cdk-1 has been used as loading control. Phospho-Cdk-1 levels normalised on total Cdk-1 were quantified and shown as fold change of WT (*n* = 8 mice per genotype). **d** Representative immunoblotting of p-Drp1-S616 in gastrocnemius lysates from WT and Drp/MC mice at P7. Total Drp1 has been used as loading control. Phospho-Drp1 levels normalised on total Drp1 were quantified and shown as fold change of WT (*n* = 5 mice per genotype). Values are expressed as mean ± SEM. * vs WT mice (*P < 0.05, **P < 0.01, ***P < 0.001).

Consistently with this, immunoblot analysis revealed a significant increase in desmin protein levels in Drp/MC muscle at P100 (Fig. 5d), while at P25 desmin expression was unchanged (Fig. S5B). In line with the presence of aggregates, an ~200 kDa band was detected specifically in Drp1 overexpressing muscle, representing detergent-resistant tetramers, i.e. a desmin insoluble fraction (Fig. 5e). Desmin is subjected to many post-translational modifications that cause disassembly of its network [42] and in agreement with this, we detected high phosphorylation of desmin at Ser-31 in Drp/MC muscle at P25 (Fig. 6a). We also carried out a time-course analysis and we found phospho-Ser-31 positive aggregates at P7 that were evident at P25 and P100 as well and were localised in the subsarcolemmal region and in the centre of the fibres (Fig. 6b). Cyclin-dependent kinase-1 (Cdk-1) is responsible for this phosphorylation [43]: consistently, it was more active in Drp/MC muscle at P7 (Fig. 6c). Along with desmin, also Drp1 was phosphorylated at both P7 (Fig. 6d) and P25 (Fig. 1a) in keeping with the notion that Cdk-1 also drives the phosphorylation of Drp1 at Ser-616 [32]. These results indicate that the higher activity of Cdk-1 in Drp/MC mice leads to persistent desmin and Drp1 phosphorylations that contribute to muscle phenotype.

**Fig. 7:**
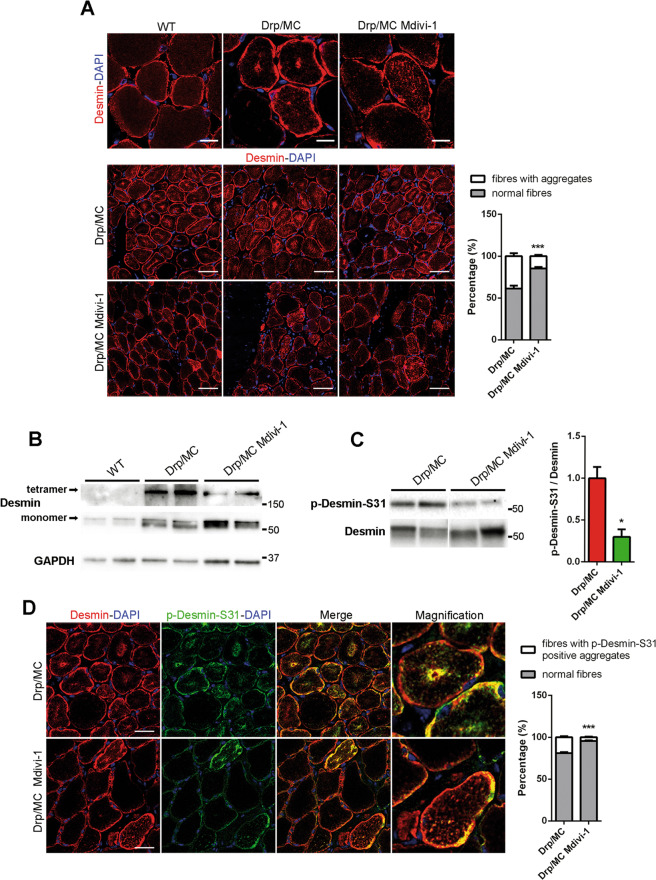
Mdivi-1 treatment improves desmin network abnormalities in Drp/MC mice. **a** Representative desmin immunostaining (red) of TA sections from WT and Drp/MC mice treated or not with Mdivi-1. DAPI is used as nuclear staining (blue) (upper panel scale bars = 10 μm; lower panel scale bars = 50 μm). Fibres containing desmin clusters were quantified and shown as percentage of total fibres (*n* = 3 mice per condition). **b** Representative immunoblotting of desmin in TA lysates from WT and Drp/MC mice treated or not with Mdivi-1. Both desmin tetramers and monomers signals are shown. GAPDH has been used as a loading control. **c** Representative immunoblotting of p-Desmin-S31 in gastrocnemius lysates from Drp/MC mice treated or not with Mdivi-1. Total desmin has been used as loading control. Phospho-desmin levels normalised on total desmin were quantified and shown as fold change of Drp/MC (*n* ≥ 3 mice per genotype). **d** Representative p-Desmin-S31 (green) and desmin (red) immunostainings of TA sections from Drp/MC mice treated or not with Mdivi-1. DAPI is used as nuclear staining (blue) (scale bar = 25 μm). Fibres containing phospho-desmin aggregates were quantified and shown as percentage of total fibres (*n* = 3 mice per condition). Values are expressed as mean ± SEM. * vs untreated Drp/MC mice (*P < 0.05, ***P < 0.001).

After Mdivi-1 treatment, desmin pattern appeared improved compared with untreated Drp/MC mice, with less sarcoplasmic aggregates, even if its shape did not fully recover to match that of WT (Fig. 7a). Consistently the presence of insoluble tetramers was reduced (Fig. 7b) as well as the phosphorylation of desmin at Ser-31 (Fig. 7c) with single muscle fibres showing a more striated outline after Mdivi-1 treatment (Fig. S5C). These findings were superimposable with the partial recovery of the mitochondrial network induced by Mdivi-1. However, the Drp1-dependent desmin aggregation was not completely blunted as shown by persisting phospho-desmin Ser-31 aggregates (Fig. 7d) and the unfolded protein response, enhanced in Drp/MC mice [15], was still active (Fig. S5D, E), thus sustaining the partial effects of Mdivi-1 administration.

**Fig. 8:**
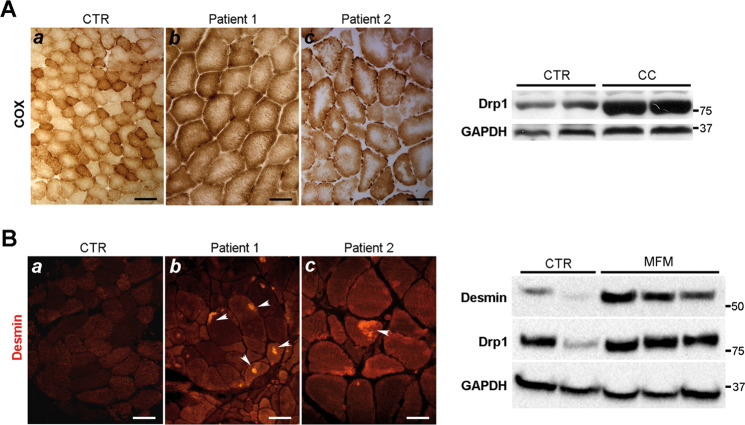
Central core and myofibrillar myopathy show high Drp1 expression. **a** Central Core (CC) myopathy left panel: COX staining in two human muscle samples showing central area without or reduced COX activity (b–c). (a) COX activity in normal human muscle samples. Scale Bar (a: 50 µm, b–c: 25 µm). Right panel: Drp1 immunoblotting in muscle homogenates from controls and CC human biopsies. **b** Myofibrillar myopathy (MFM) left panel: desmin immunostaining showing desmin accumulation in two human muscle samples (white arrows) (b–c). (a) Desmin immunostaining in normal human muscle sample. Scale Bar (a: 40 µm, b–c: 25 µm). Right panel: Drp1 immunoblotting in muscle homogenates from controls and MFM human biopsies.

The muscular phenotype of the Drp/MC mice resembles that observed in the central core disease, characterised by core areas without COX activity (Fig. 8a, left panel), and the myofibrillar myopathy characterised by desmin accumulation and aggregation (Fig. 8b, left panel). Because of this similarity, we analysed human muscle biopsies from patients affected by central core disease or myofibrillar myopathy. In both conditions Drp1 was overexpressed compared with healthy controls (Fig. 8a, b, right panels), suggesting a possible role of Drp1 in establishing the phenotype.

## Discussion

Using a specific skeletal muscle transgenic model we report on a hitherto unknown function of Drp1, i.e. its role in controlling both mitochondrial repositioning and desmin assembling.

Defects in desmin assembly or desmin loss cause subsarcolemmal mitochondria clumping [12, 44–46]. This phenomenon occurs in Drp/MC mice leading to fibres characterised by focal depletion of mitochondria from their centre. In Drp/MC mice desmin does not have its typical striated pattern, being aggregated in the subsarcolemmal region and accumulated in mitochondria rubbed-out areas, resembling the pattern observed in some myofibrillar myopathies [45, 47]. The presence of insoluble tetramers may be explained by an inefficient desmin assembling leading to the collapse and aggregation of filaments [44]. It is well established that IF are dynamic structures and reversible phosphorylation is a mechanism that plausibly maintains this dynamism affecting its assembling and disassembling [48, 49]. In line with this, we found that in Drp/MC mice desmin was hyperphosphorylated in Ser-31 by a hyperactivated Cdk-1. This led to persistent phosphorylation of both desmin and Drp1 and resulted in desmin disassembling and Drp1 activation. Desmin preserves mitochondria in close proximity to myofibrils and promotes the transfer of the energy. It is able to sense and respond to stress regulating many processes including the gene expression, thus desmin can be considered a mechanosensor and transducer of signals to the nucleus [42, 48, 50]. Moreover, desmin disruption compromises the cross-talk between mitochondria and other organelles affecting mitochondrial biogenesis and function [48]. At P7 we observed induction of fission and desmin phospho-Ser-31 positive aggregates, while the mitochondrial repositioning, with subsarcolemmal clumping, and the impaired mitochondrial biogenesis occurred at P25 in Drp/MC mice. This suggests that an early desmin disruption could compromise the mechanotransduction system affecting both the biogenesis and the maintenance of the fissioned-mitochondria in the proper position, driving upregulation of kinesin-1 and mitochondrial repositioning at sarcolemma.

The investigations on desmin alterations allowed us to uncover the direct action of Drp1 on mitochondrial distribution. Mitochondrial motility is a feature of many eukaryotic cells and this motility permits the mitochondria to be distributed in view of local energy use. In neurons as well as in muscle, much of this motility is MT-based and requires kinesins as motor proteins [8, 39]. Kinesin-1 is a tetramer consisting of kinesin heavy chains and kinesin light chains [18] with the heavy chain KIF5B primarily involved in the mitochondria movements along MT in the muscle [8]. In muscle, Drp1 is able to control kinesin-1 complex activation.

Drp1 promotes fission and mediates the mobilisation of mitochondria along the MT bound to KLC1; we found that the Drp1-KLC1 complex released KIF5B allowing its association with MT to transport mitochondria and increased the speed and distance they travelled individually. The MT binding assay and PLA experiments clearly established that the active form of kinesin-1 involved only KIF5B and not the tetramer, excluding situations previously described in which kinesin activation is mediated by conformational changes and association with binding partners [40, 51]. Therefore the mitochondria repositioning observed in Drp/MC mice does not require KLC1; this non-conventional kinesin heavy chain-based transport has been previously reported also in *Neurospora crassa* [52], neuronal dendrites [53], and axons [39]. Our results describe for the first time this mechanism in muscle and identify Drp1 as a new regulator of the complex in vivo. The mechanism of kinesin-1 activation is dependent on Drp1 levels, indeed we found that KIF5B-MT interaction was increased in Drp1-overexpressing condition, while it decreased, proportionally to Drp1 reduction, after Mdivi-1-treatment. However, the positive effects of Mdivi-1 were only partial because it did not restore Drp1 levels to those observed under basal conditions. Whereas the efficacy of Mdivi-1 as a Drp1 inhibitor has recently been challenged [54], several lines of evidence support this activity [41, 55, 56] showing that Mdivi-1 reduces Drp1 levels, prevents mitochondrial fragmentation, enhances fusion and promotes the formation of mitochondrial network and we confirm these findings. In agreement with mitochondrial improvements, Mdivi-1 ameliorated also desmin pattern in Drp/MC muscle, decreasing the accumulation of insoluble sarcoplasmic tetramers and consistently its phosphorylation in Ser-31. Desmin might be involved in the control of proper fusion-fission events [50]; Mdivi-1 can not prevent Cdk-1 activation that occurs at P7, rather it inhibits fission and establishes a direct link between mitochondrial fission and desmin phosphorylation. However, as Mdivi-1 did not fully restore mitochondrial network, it did not allow a full recovery of desmin assembling. These results confirm that desmin collapse in Drp/MC mice depends on its phosphorylation on Ser-31, that requires both Cdk-1 activation and mitochondrial fission. These events compromise the stability of mitochondrial network promoting its remodelling mediated by MT-dependent trafficking. We also suggest that Drp1 levels play a central role in coordinating mitochondrial repositioning, coupling to KLC1 and activating KIF5B. This unravels a hitherto unknown action of Drp1 as a modulator of organelle movements. This new mechanism could be relevant in some human conditions, such as central core disease and myofibrillar myopathy. We found that Drp1 was upregulated in both conditions suggesting its possible involvement in establishing the phenotype. The extensive characterisation of the role of Drp1 in these two muscular disorders may unravel novel pathogenic mechanisms responsible for muscular degeneration and provide possible novel therapeutic targets.

## Supplementary information

Supplementary figure and video legends

Supplementary Figure 1

Supplementary Figure 2

Supplementary Figure 3

Supplementary Figure 4

Supplementary Figure 5

Video 1

Video 2

Video 3

Video 4
