# Supplementary material for: Drp1 overexpression induces desmin disassembling and drives kinesin-1 activation promoting mitochondrial trafficking in skeletal muscle
Source: Cell Death Differ. 2020 Feb 10;27(8):2383–401. doi: 10.1038/s41418-020-0510-7 (PMC7370230; doi:10.1038/s41418-020-0510-7)
Supplement: Supplementary file 1 — Supplementary figure and video legends [file 41418_2020_510_MOESM1_ESM.docx]

**Supplementary figure legends**

**Supplementary Figure 1:** **Drp1 overexpression affects kinesin-1 complex expression.**

**(A-C)** KIF5B and KLC1 immunoblots using TA lysates from WT and Drp/MC mice at P1, P7 (A), P25 (B), and P100 (C). GAPDH has been used as a loading control. Protein levels were quantified and shown as fold change of WT (n ≥ 5 mice per genotype). **(D)** RT-qPCR analysis of KIF5B and KLC1 expression in TA at P25 and P100 (n ≥ 5 mice per genotype). Values are expressed as mean ± SEM. * *vs* WT (** P < 0.01).

**Supplementary Figure 2. Effects of Drp1 on mitochondrial network and movements in myotubes**

**(A)** Confocal images of the mitochondrial network (green) in WT/PhAM and Drp/MC/PhAM myotubes. DAPI nuclear counterstaining (blue) is provided (scale bar = 25 μm). Magnifications in lower panels showed the mitochondrial network structure by Skeletonize (2D/3D) ImageJ software plugin. Mitochondria branch length distribution, number of branches per area (values expressed as mean ± SEM), and elongation coefficient are calculated (n = 3 independent experiments). **(B)** Stills from movies showing mitochondrial network (green) movements in WT/PhAM and Drp/MC/PhAM myotubes. Arrowheads indicate mitochondrial movements during the time (scale bar = 5 µm). * *vs* WT (* P < 0.05, ** P < 0.01, *** P < 0.001).

**Supplementary Figure 3. Effects of Drp1 on cytoskeleton and MT-dependent transport.**

**(A-B)** Representative tubulin and actin immunostaining (red) of TA single fibres from WT and Drp/MC mice at P25 (A) and P100 (B) (scale bar = 10 μm). **(C)** Stills from movies showing mitochondrial network movements in WT/PhAM and Drp/MC/PhAM myotubes. Arrowheads indicate mitochondrial movements during the time with representative mitochondria distance travelled highlighted (scale bar = 5 µm). **(D)** Representative immunoblotting of KLC1 in mitochondrial and cytosolic fractions of TA from WT and Drp/MC mice at P25. COX IV and GAPDH have been used as mitochondrial and cytosolic markers respectively. **(E)** Representative negative KLC1:α-Tubulin PLA (no red puncta) on TA muscle sections from WT and Drp/MC mice at P25. DAPI nuclear counterstaining (blue) is provided (scale bar = 25 µm).

**Supplementary Figure 4. Mdivi-1 restores mitochondrial architecture in Drp/MC myotubes**

**(A)** Magnified confocal 3D reconstructions of TA fibres from WT/PhAM and Drp/MC/PhAM mice treated or not with Mdivi-1 (scale bar = 5 µm). **(B)** Average mitochondrial branch length quantification (n ≥ 3 mice per condition). * *vs* Drp/MC (** P < 0.01). **(C)** qPCR analysis of mtDNA content in TA from Drp/MC mice treated or not with Mdivi-1. Data are shown as fold change of untreated Drp/MC (n ≥ 3 mice per condition). Values are expressed as mean ± SEM. **(D)** Representative immunoblotting of KLC1 and KIF5B in muscle homogenates from TA of Drp/MC mice treated or not with Mdivi-1. GAPDH has been used as loading control**. (E)** Representative images of the mitochondrial network in Drp/MC/PhAM myotubes treated or not with Mdivi-1. DAPI nuclear counterstaining (blue) is provided (scale bar = 25 μm). Magnifications in lower panels show the mitochondrial network structure by Skeletonize (2D/3D) ImageJ software plugin. Mitochondria branch length distribution, number of branches per area (values expressed as mean ± SEM) and elongation coefficient are calculated (n = 3 independent experiments, n ≥ 20 myotubes). * *vs* Drp/MC (* P < 0.05, ** P < 0.01 *** P < 0.001). **(F)** Representative immunoblotting of Drp1, KIF5B and KLC1 in lysates of Drp/MC myotubes treated or not with Mdivi-1. GAPDH has been used as a loading control. **(G)** Representative PLA Drp1:KLC1 (red puncta) on TA single fibres from Drp/MC mice treated or not with Mdivi-1. (scale bar = 25 µm). PLA puncta were quantified and shown as a percentage relative to untreated Drp/MC mice (n = 3 mice per condition). **(H)** Representative PLA KIF5B:α-Tubulin (red puncta) on TA single fibres from Drp/MC mice treated or not with Mdivi-1. (scale bar = 25 µm). PLA puncta were quantified and shown as a percentage relative to untreated Drp/MC mice (n = 3 mice per condition). (G-H) * *vs* Drp/MC (** P < 0.01). Values are expressed as mean ± SEM

**Supplementary Figure 5. Desmin pattern and UPR response in Drp/MC mice**

**(A)** Representative desmin immunostaining (red) of TA single fibres from WT/PhAM and Drp/MC/PhAM mice at P25. Mitochondrial network (green) images are provided (scale bar = 10 μm). **(B)** Representative immunoblotting of desmin in TA lysates from WT and Drp/MC mice at P25. GAPDH has been used as a loading control. **(C)** Representative desmin immunostaining (red) of TA single fibres from WT and Drp/MC mice treated or not with Mdivi-1 (scale bar = 10 μm). **(D)** Representative immunoblotting of phospho-eIF2α on serine 51 (p-eIF2α-S51), total eIF2α, and Clpp in TA lysates from WT and Drp/MC mice treated or not with Mdivi-1. GAPDH has been used as a loading control. p-eIF2α protein levels normalized on total eIF2α and Clpp protein levels were quantified and shown as fold change of WT (n ≥ 3 mice per condition). **(E)** RT-qPCR analysis of stress-responsive genes in TA from WT, Drp/MC and Mdivi-1-treated Drp/MC mice. Data are shown as fold change of WT (n ≥ 3 mice per condition). (D-E) Values are expressed as mean ± SEM. * *vs* WT (* P < 0.05, ** P < 0.01, *** P < 0.001).

**Supplementary video legends**

**Video 1 Mitochondrial movements increase in Drp/MC/PhAM mice**

WT/PhAM (upper movie) and Drp/MC/PhAM (lower movie) Tibialis Anterior muscle were photoconverted using the 405nm laser. GFP and mCherry signals were excited respectively with a 488nm and 561 nm 50mW diode lasers. Muscles were maintained in a humidified chamber with constant temperature (37°C) and CO2 (5%). Images were taken every 2 sec from 10 min. Time-lapse movies are shown at a frame rate of 150 fps.

**Video 2.** **Mitochondrial displacement increases in Drp/MC/PhAM myotubes**

Myotubes from WT (upper movie) Drp/MC/PhAM (lower movie) mice were obtained after 48h of satellite cells differentiation in μ-slide 8-well (ibiTreat, Ibidi, Kat.Nr. 80826). Myotubes were maintained in a humidified chamber with constant temperature (37°C) and CO2 (5%). Images were taken every 10 sec from 10 min. Time-lapse movies are shown at a frame rate of 15 fps.

**Video 3. Mitochondria in Drp/MC myotubes cover a longer distance**

Myotubes from WT (upper movie) Drp/MC/PhAM (lower movie) mice were obtained after 48h of satellite cells differentiation in μ-slide 8-well (ibiTreat, Ibidi, Kat.Nr. 80826). Myotubes were maintained in a humidified chamber with constant temperature (37°C) and CO2 (5%). Images were taken every 10 sec from 10 min. Time-lapse movies are shown at a frame rate of 15 fps.

**Video 4. Mitochondrial displacement decreases after Mdivi-1 treatment of myotubes**

Myotubes from Drp/MC/PhAM mice treated (lower movie) or not (upper movie) with Mdivi-1 for 24h in μ-slide 8-well (ibiTreat, Ibidi, Kat.Nr. 80826). Myotubes were maintained in a humidified chamber with constant temperature (37°C) and CO2 (5%). Images were taken every 10 sec from 10 min. Time-lapse movies are shown at a frame rate of 15 fps.
