# Supplementary figures and images for: Drp1 overexpression induces desmin disassembling and drives kinesin-1 activation promoting mitochondrial trafficking in skeletal muscle
Source: Cell Death Differ. 2020 Feb 10;27(8):2383–401. doi: 10.1038/s41418-020-0510-7 (PMC7370230; doi:10.1038/s41418-020-0510-7)

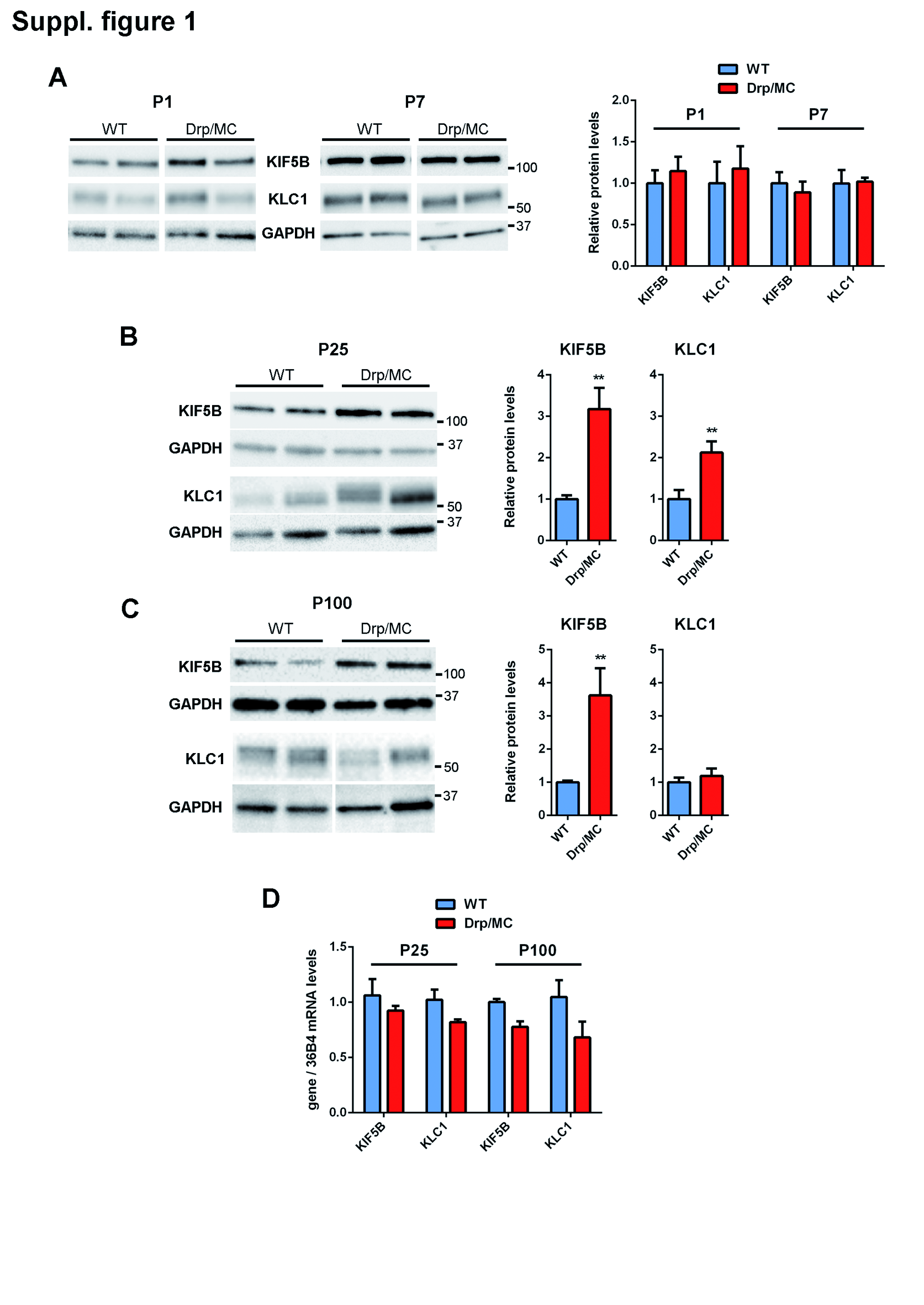

Supplement: Supplementary file 2 — Supplementary Figure 1 [file 41418_2020_510_MOESM2_ESM.tif]

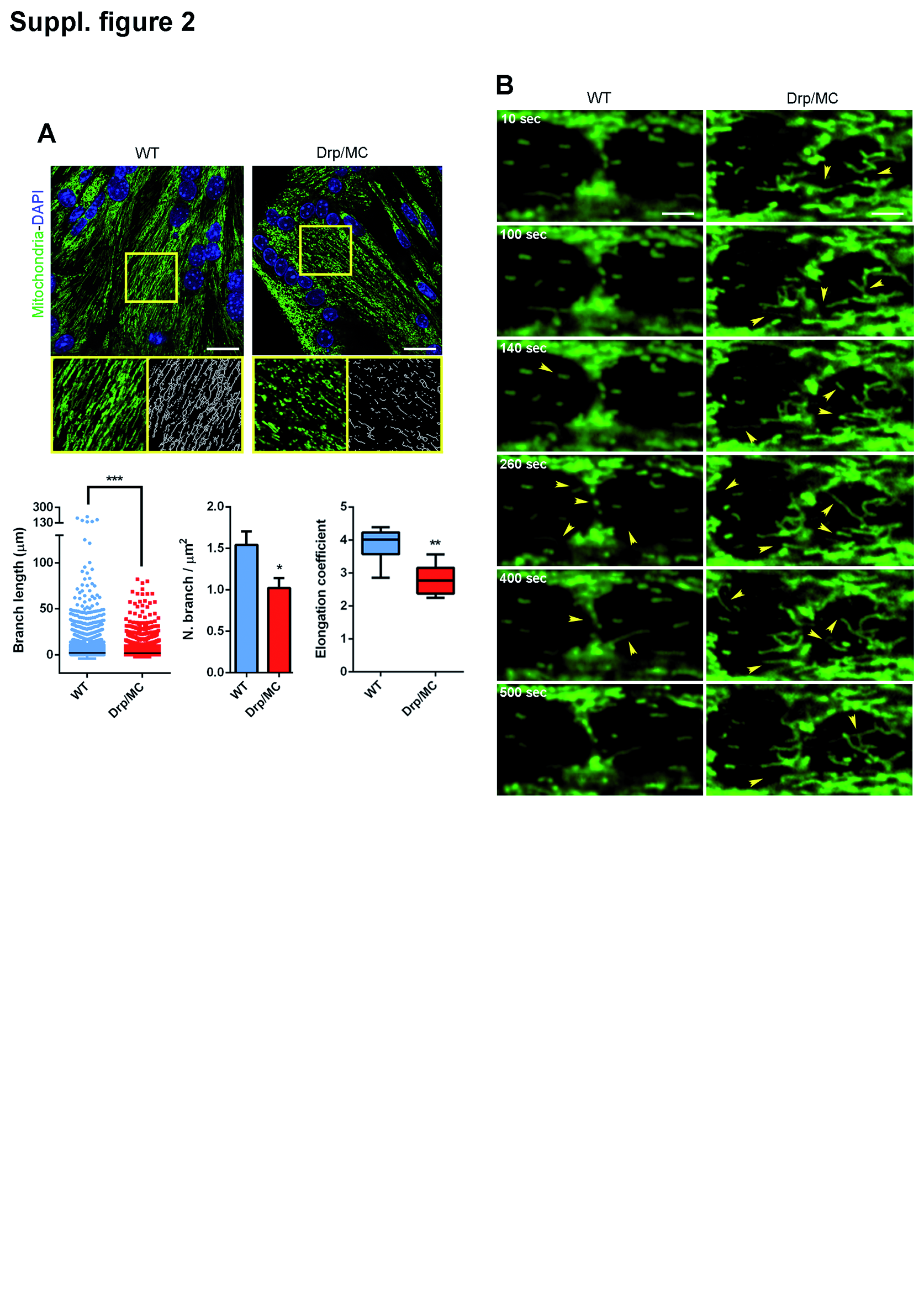

Supplement: Supplementary file 3 — Supplementary Figure 2 [file 41418_2020_510_MOESM3_ESM.tif]

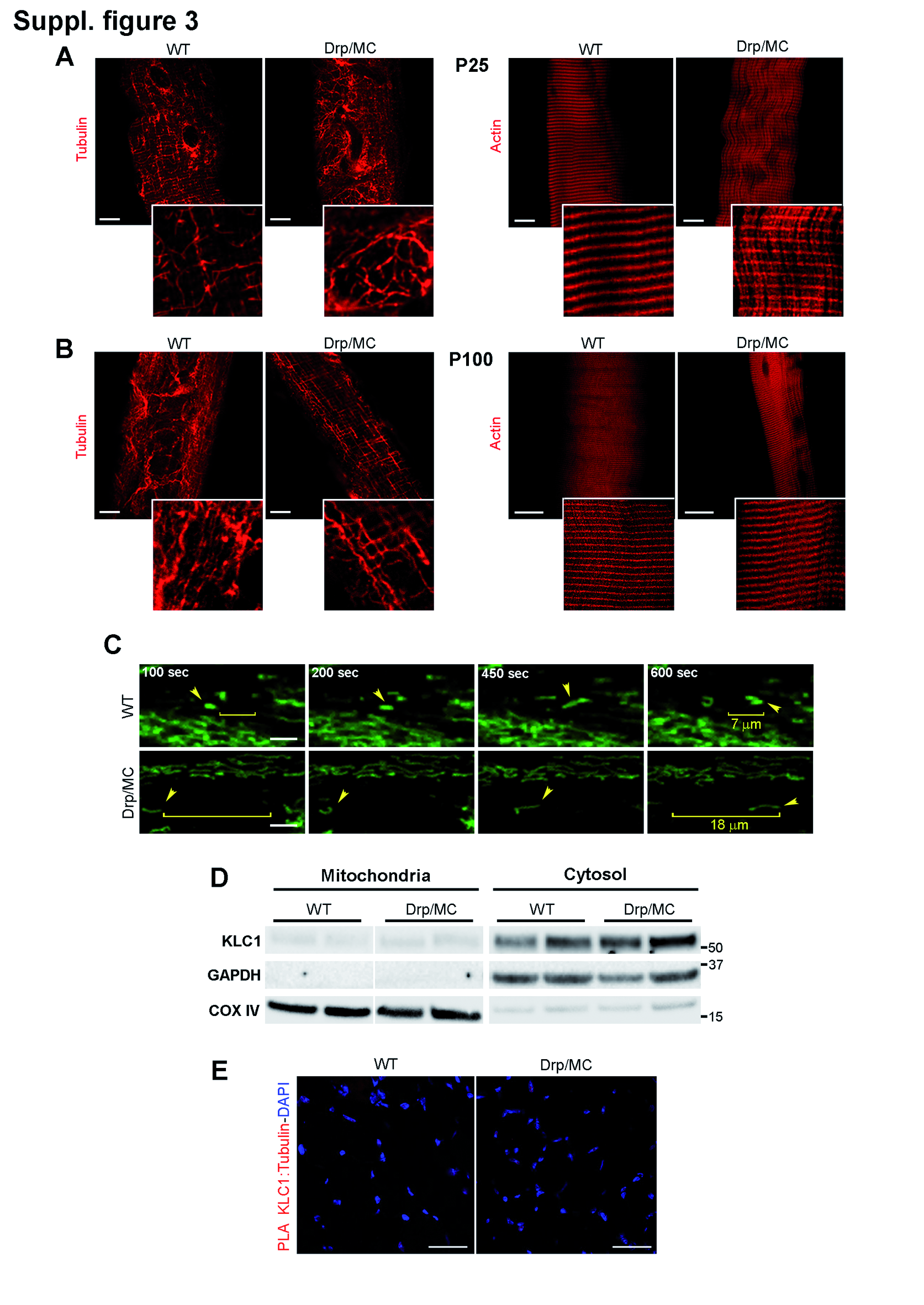

Supplement: Supplementary file 4 — Supplementary Figure 3 [file 41418_2020_510_MOESM4_ESM.tif]

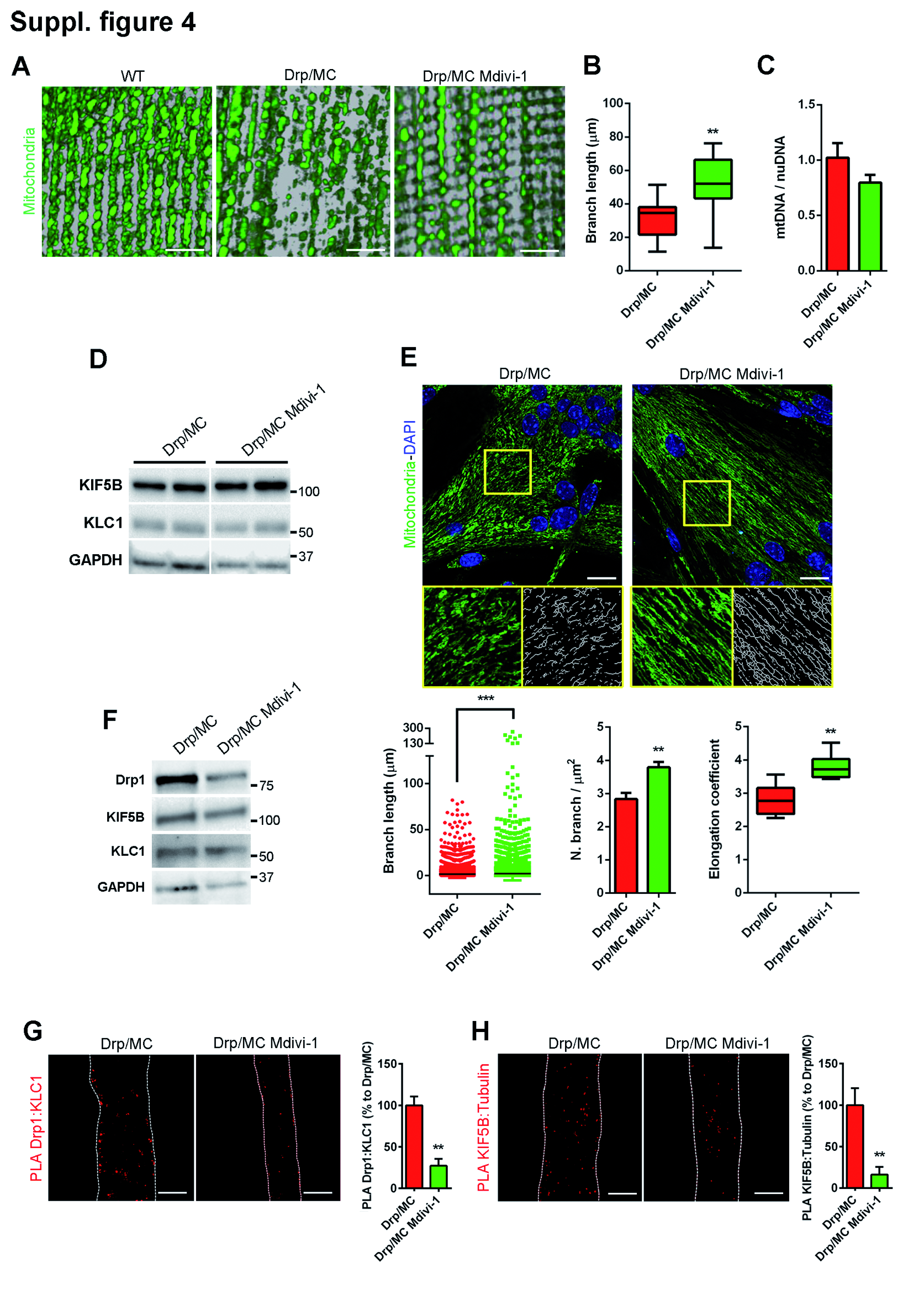

Supplement: Supplementary file 5 — Supplementary Figure 4 [file 41418_2020_510_MOESM5_ESM.tif]

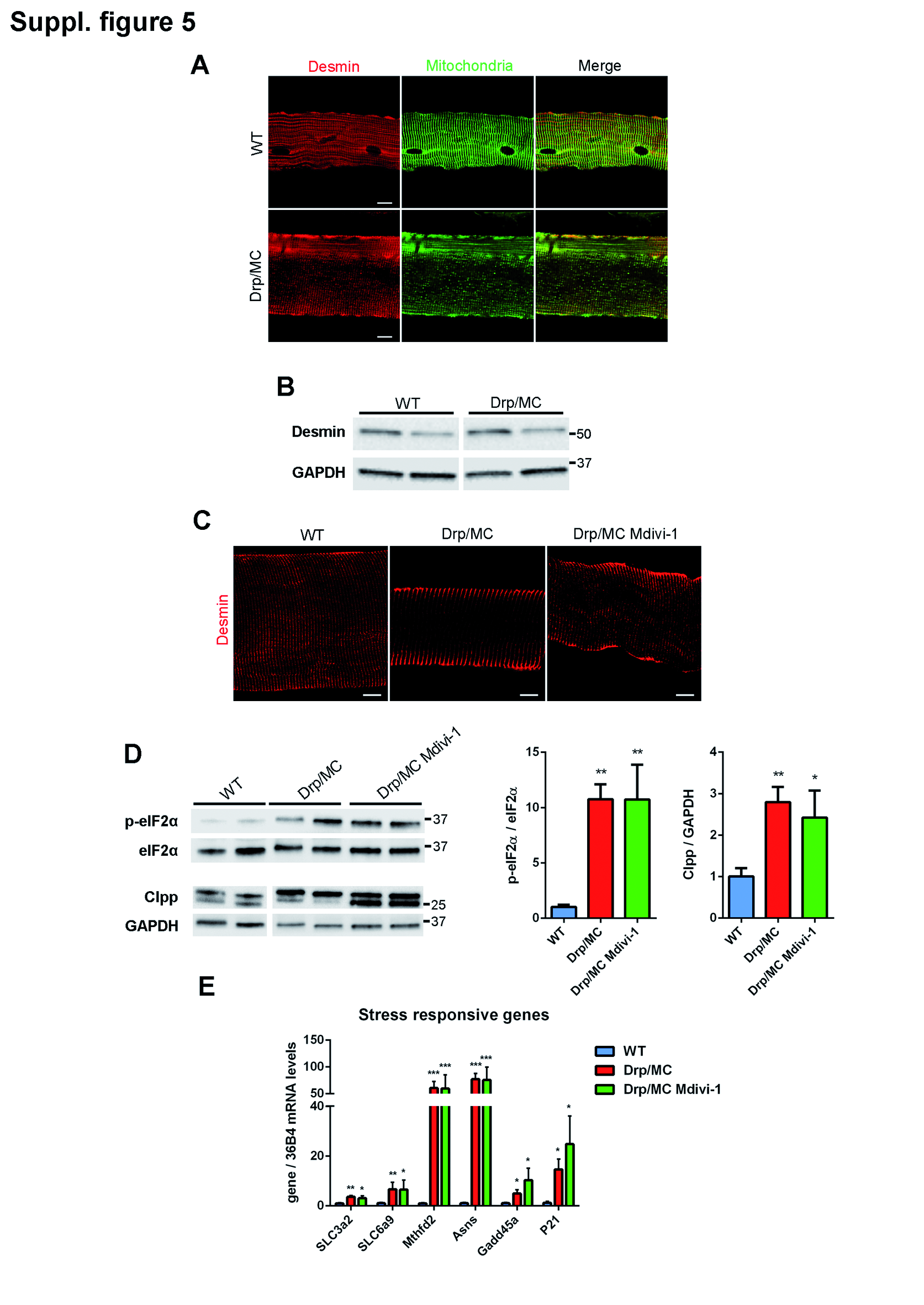

Supplement: Supplementary file 6 — Supplementary Figure 5 [file 41418_2020_510_MOESM6_ESM.tif]
